# Ova-looking feminist theory: a call for consideration within health professions education and research

**DOI:** 10.1007/s10459-022-10108-8

**Published:** 2022-04-07

**Authors:** G. M. Finn, M. E. L. Brown

**Affiliations:** 1 School of Medical Sciences, Faculty of Biology, Medicine and Health, The University of Manchester, Manchester, UK; 2 Medical Education Innovation and Research Centre, Imperial College London, London, UK

**Keywords:** Gender, Feminism, Curriculum, Research, Health inequalities, Health professions, Education

## Abstract

The role of feminist theory in health professions education is often ‘ova-looked’. Gender is one cause of healthcare inequalities within contemporary medicine. Shockingly, according to the World Health Organisation, no European member state has achieved full gender equity in regard to health outcomes. Further, contemporary curricula have not evolved to reflect the realities of a diverse society that remains riddled with inequity. This paper outlines the history of feminist theory, and applies it to health professions education research and teaching, in order to advocate for its continued relevance within contemporary healthcare.

## Introduction

This paper outlines the history of feminist theory, and applies it to health professions education research and teaching, to advocate for its continued relevance within contemporary healthcare. Sharma presented an overview of feminist theory in a 2019 publication. In this paper we consider the history in order to take a deeper dive into several feminist theories, each selected purposively from a different ‘wave’ of feminism and contextualise them within health professions education. We consider pertinent literature in order to offer practical implications and demonstrate the value added for our field. The theories presented are not exhaustive. Rather, we have selected those that offer a new lens through which to consider our field, with some choices informed by our own experiences as medical educators, and others to reflect the spectrum of health professions education research, scholarship, and teaching. We have highlighted areas where future work is warranted within our discipline. In particular, we noted absence, as defined by Paton and colleagues (2020) as: absence of content, absence of research, and absence of evidence. However, where we see a gap, others may see different absences, or perhaps across different domains of our field such as within a curricular space as opposed to research. As Malorie Blackman said, **“** *Reading is an exercise in empathy; an exercise in walking in someone else’s shoes for a while.* (Blackman, 2021)” Thus, we urge readers to continue their journey and discover the implications of feminist theory for themselves and the students, educators, and patients who they accompany along the road.

## What is feminism?

A critique often advanced against feminist scholars is that they are unable, or unwilling (Thompson, 2001) to offer a clear, or simple answer to the question ‘What is feminism?’, or ‘What is feminist theory’ (Smelser and Baltes, 2001). Feminist theory represents a diverse range of concepts, theories and principles. Maggie Humm (2015) has variously described feminism as ‘equal rights for women’, ‘the ideology of women’s liberation’, as ‘the theory of the woman’s point of view’, and ‘fundamentally about women’s experience’(Thompson, 2001). It is this multiplicity that makes feminism such an important and flexible topic, but frustrating or alienating for others. As a field rooted in positivist and postpositivist ways of thinking which maintain the existence of a singular, objective reality, the inability to succinctly summarise feminism as one, neat way of viewing the world is liable to criticism from some. Yet, the diverse range of theories captured within the umbrella of feminism offers a flexibility to approaching educational or research questions that can more readily expose and remedy gender inequities. Whilst we cannot claim to offer a definitive and enduring answer to the broad question of ‘What is feminism’, this paper aims to highlight the core features which span several feminist approaches to education and research, applying these ideas to the field of health professions education to explore ways in which the feminist agenda could be advanced in practice.

## Why should health professions educators care about feminism?

Gender is one cause of healthcare inequalities within contemporary medicine. Shockingly, according to the World Health Organisation (2020), no European member state has achieved full gender equity in regard to health outcomes. Within the UK alone, it is estimated that there are two preventable gender gap-related deaths per day (Royal College of Obstetrics and Gynaecologists., 2019). Gender inequality affects the working lives of women, too. Indeed, recent evidence demonstrates that women are still not treated equally within health professions education, from students (Brown et al., 2020) across the career trajectory (Finn and Morgan, 2020). For a field responsible for training tomorrow’s clinicians, who will go on to hold great power to influence health-related inequalities, a lack of gender equality within the educational and research domains of our field should be of supreme concern. Yet, far too often, gender equity is dismissed as a women’s issue, and feminism as a position of the fervent, and extreme. The stigma associated with identifying oneself as a feminist has stalled the critical evaluation of the long-accepted systems and structures in place in many academic fields which is necessary to advance the cause of justice and equality for women. Health professions education, unfortunately, is no exception. So commonplace is the inequity and discrimination faced by women, the British Medical Journal published a lexicon of terms used in association with women and gender bias. Examples include ova-looked, femi-nazi, mansplaining and repriwomand (Choo and DeMayo, 2018).

Some argue that we are now in the ‘post-feminism’ era, characterised by the perception that feminism is either over, or no longer relevant (Leavy and Harris, 2018). We disagree – while healthcare services have undergone a renaissance, with greater awareness of sex, gender, and associated health inequalities, health professions curricula have not kept pace (Finn et al., 2021). Though feminism and feminist theory have evolved and increasingly consider individuals holistically, and as embedded in iniquitous social structures, there is more work to be done to advance feminist thinking and advocacy in our field.

Firstly, inequality pervades the career paths of health professions educators, and of medics. The struggles faced by women in workforces are well documented (Williams, 2005; Williams et al., 2006; Williams and Dempsey, 2014), and health professions education is no exception (Varpio et al., 2020a; Brown et al., 2020; Finn and Morgan, 2020). As with the aforementioned lexicon of gender biases (Choo and DeMayo, 2018), a plethora of metaphors pertaining to the differential experiences of women’s career trajectories exist, further evidencing the inequity of experience. The most common metaphors include the sticky floor, the glass ceiling, and the labyrinth (Tesch et al., 1995; Yap and Konrad, 2009; Kark and Eagly, 2010). Within health professions education, Varpio and colleagues (2020a) described the arduous path for women in achieving their professorial positions, when compared to men. Maternal status was cited as impacting upon the careers of the participants in Varpio’s study, further supported by explicit examples of maternal wall bias within studies by Finn (2020) and Brown (2020). So ingrained and systemic is the inequity, that within the United Kingdom, initiatives such as Athena SWAN have been developed. Athena SWAN is a charter recognises good practices in higher education and research institutions towards the advancement of gender equality, and, more recently, towards broader equality for marginalised groups (Ovseiko et al., 2017).

Given that education lays the foundational building blocks for future physicians’ and educators’ attitudes and perceptions, health professions curricula are ripe for feminist change. Even in 2021, curricula, organisational culture, physical education environments remain binary in regard to gender. Dualistic thinking prevails, where the underlying assumptions of an education or healthcare system comprised of only two contrasting, mutually exclusive choices or realities remain the accepted reality. Recent studies (Brown et al., 2020; Finn and Morgan., 2020) reported that both medical students and faculty were recipients of tacit gender bias messaging from their physical environments. Such messages served to manifest organisational values regarding who is most welcome, again presenting a gendered message through imagery, for example, that remains ‘too male, too pale…too stale’. Further, within curricula, marginalised groups, for example the LGBTQIA + community, are underrepresented; this poses the risk of potentially perpetuating the well-documented health inequalities experienced by LGBTQIA + individuals and their community (Finn et al., 2021). Sex and gender are often conflated, with many curricula failing to integrate these issues into their teaching and learning. Sex and gender are fundamental principles for healthcare given that differences exist between them with respect to the manifestation and prevalence of many conditions and diseases (Finn et al., 2022) (Miller et al., 2012). Failure to differentiate between sex and gender has been highlighted as problematic in curriculum design, particularly within anatomy (Lazarus & Sanchez., 2021).

Additionally, as gendered inequities in healthcare continue globally, including in women’s health and reproductive decision making, awareness of feminist theory is pivotal in engaging students in meaningful discourse with regard to social determinants of health. It is the duty of healthcare educators to ensure that students are aware that the ‘gender relations of power constitute the root causes of gender inequality and are among the most influential of the social determinants of health’ (Sen and Östlin, 2008.)

Contemporary health professions education, such as during the COVID-19 pandemic, provides further evidence of the gendered inequity within global healthcare systems and further challenges the notion that feminism is no longer relevant. The differential impact of the pandemic on men and women has been well documented (Fortier, 2020; Ewing-Nelson, 2020; Froldi and Dorigo, 2020), in terms of the socio-economic hardships, gendered-divisions of labour and wellbeing. The clinical academic workforce, namely the educators doing the teaching and research, are one group where differential impacts have been frequently reported (Finn and Morgan, 2020). Finally, the disease itself has notable risk factors associated with worse outcomes for patients including protected characteristics^1^ such as older age and male gender (Froldi and Dorigo, 2020), further evidencing the need for gender theory within curriculum design.

Gendered assumptions are documented as stagnating consultations with patients, particularly heteronormative assumptions (e.g., by negatively influencing rapport or preventing health seeking behaviours for sexual health issues) (Finn et al., 2021; Laughey et al., 2018; Finn et al., 2022). It is worth noting the typical manifestation of heteronormativity within curricula, as well as the curricula presentation of sex and gender (Lazarus and Sanchez, 2021). Feminist theory is crucial in challenging the gendered nature of curricula. Whitehead et al. (2012), advocate for recognition of the importance of theory-informed curricula. While caution must be paid to claiming that curricula can fix health inequalities, thus ‘diverting attention from structural social inequities’. Hubris has been cited as having led to such claims, and warnings issued that to make such claims that curricula can transform trainees or healthcare systems is arrogant (Whitehead et al., 2012). While it may be somewhat of a leap, tackling health inequalities must start somewhere and perhaps the classroom is no worse than any other place – the future healthcare workforce are also our future policy makers, after all.

## What is feminist theory?

This section offers an overview of some of the many feminist approaches to education and research. We do so not in the hope of advancing a definitive typology, for such a task we believe to be impossible and one which would always be incomplete in some way, but rather to offer a taste of some of the many approaches to feminism we see as bearing relevance to our field. Though, as we have discussed, a consensus definition of feminism does not exist, a broad explanation of what we mean when we use the term ‘feminism’ is necessary to allow readers to transparently interpret the observations and recommendations we make herein. We base our observations and analysis of feminist theories within our field on hook’s broad, encompassing definition of feminism as a ‘movement to end sexism, sexist exploitation and oppression’ (hooks, 2000).

In order to appreciate the context of the feminist theories we outline and evaluate within this paper, a brief overview of the history of feminism is necessary. Evolutions in feminist thought can be broadly mapped through consideration of feminist ‘waves’- the first wave, second wave, third wave and the fourth wave. The metaphor of ‘waves’ to describe evolutions in feminist thought is contested, with some arguing it implies a unified phenomenon of feminism, that peaks and recedes through history, imagery which is inaccurate politically and historically (Nicholson, 2010). Despite such concerns, the metaphor of ‘waves’ continues to be used widely and so, we feel, given the proviso that this term is contested and likely less-than-ideal, that it is appropriate to discuss the meanings associated with the variety of feminist waves visible within academic scholarship.

**Fig. 1:**
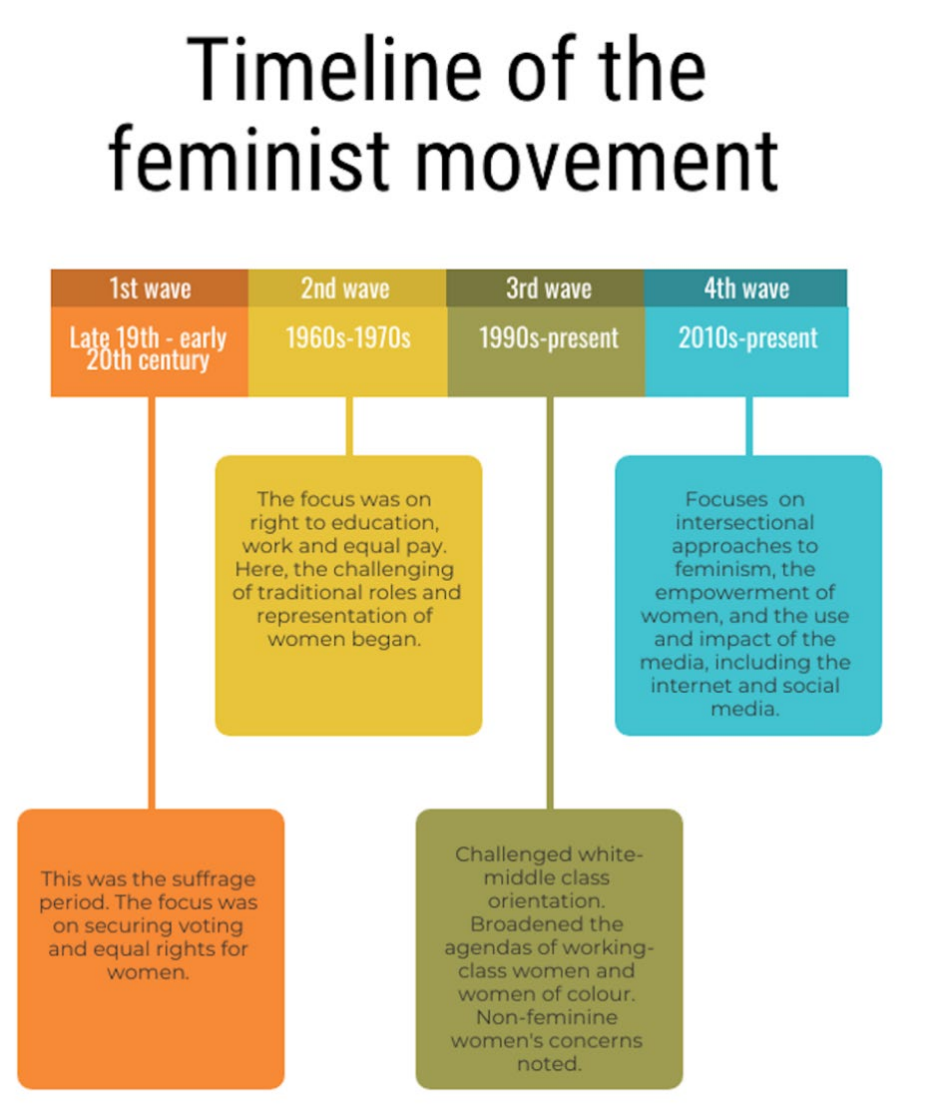
Timeline of the feminist movement (Leavy and Harris, 2018). *N.B. Interpretations of post-feminism (post-third wave feminism) and fourth wave differ

Broadly speaking, the first wave of feminism was concerned with suffrage, the second wave with issues of educational and occupational equality whilst encouraging women to consider the experience of being a woman, the third wave with a broadening of the feminist agenda to represent a more diverse group of women, and the fourth wave with highlighting the need for an intersectional approach. A timeline of the feminist movement is summarised within Fig. 1, below. It is important for the readers of this article to be aware that interpretations of feminism, specifically post-feminism (post-third wave feminism) and fourth wave differ.

As the feminist movement developed, a multitude of feminist theories sprung forth from the advances made within each wave. In their scoping review scrutinising the application of feminist theory to medical education, Sharma (2019) summarises a diverse range of feminist theoretical positions relevant to the health professions, including: liberal feminism; cultural feminism; queer feminism; anti-racist feminism; radical feminism; socialist feminism; postmodern feminism; indigenous feminism; Marxist feminism; postcolonial feminism; and intersectional feminism. These approaches to feminism collate feminist theories and unite them on the basis of the way in which they view the world (their epistemology and ontology), and the priorities they hold most dear. It is beyond the scope of this commentary to detail each of these theoretical positions in the degree of depth they each deserve. As such, we have selected a prominent and, we feel, relevant feminist approach to medical education from each wave of feminism to consider in more depth. In doing so, we add depth to some of the approaches discussed by Sharma, and consider their relevance to the field of health professions education.

**Fig. 2:**
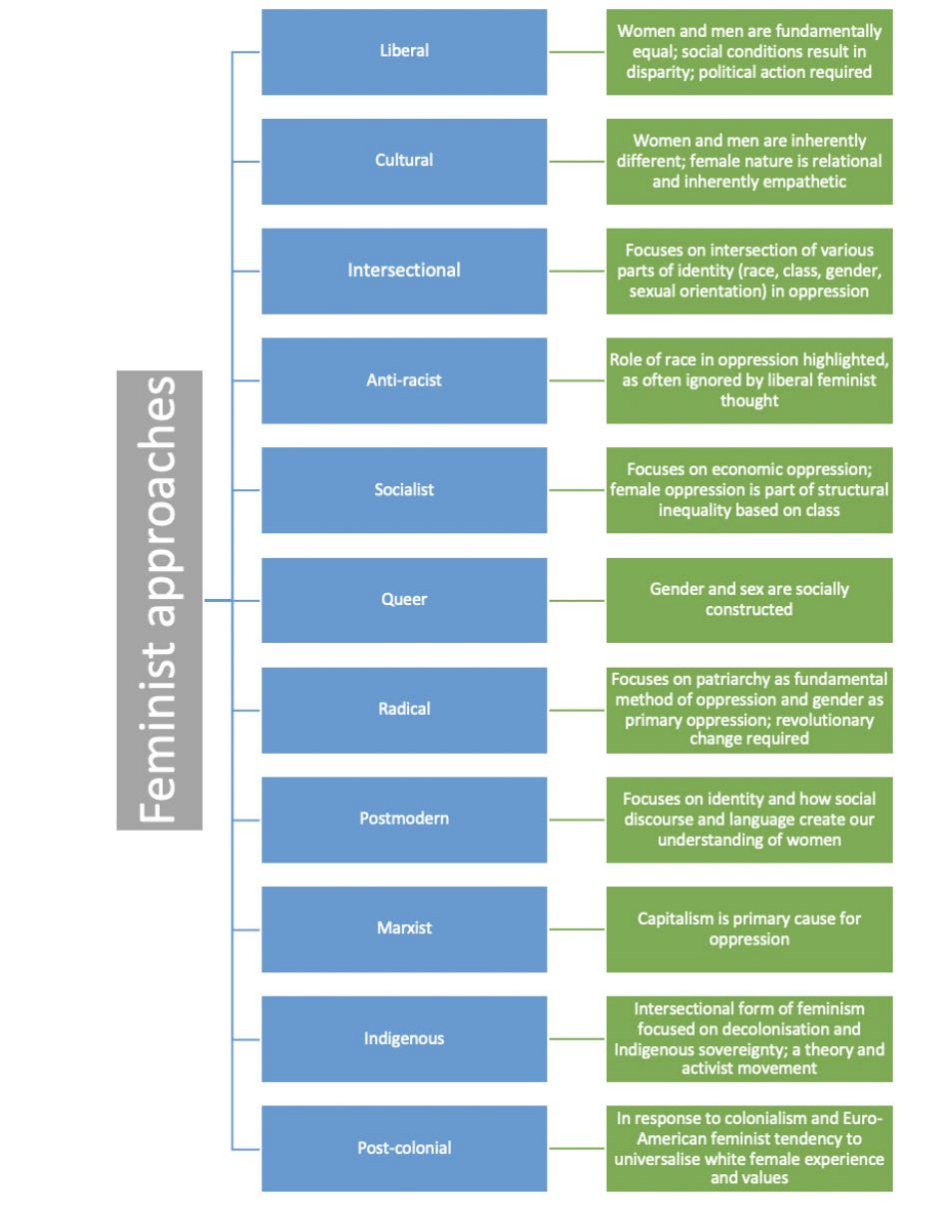
An overview of the feminist approaches identified as relevant to health professions education in Sharma’s (2019) scoping review

### 1st wave: Liberal feminism

#### Description

Liberal feminism has attracted much mainstream attention in the Western world. It originated with the plight for white, middle- and upper-class women’s equality within the suffrage movement. It is a family of theories which are united by a particular way of viewing the world. At its core, liberal feminism is concerned with the value of freedom, and the responsibility of the state to ensure freedom for women. For liberal feminists, political action is necessary to reduce inequality. Though this may sound noble, liberal feminism is held by many to be exclusionary – concerned only with freedom for white, and often only for middle- and upper-class women – indeed, it is sometimes referred to as ‘bourgeois feminism’ by more working-class movements such as socialist and Marxist feminism (Voet & Voet, 1998, Artwińska, 2020).

We have selected liberal feminism as, not only is it prominent at large (Artwińska, 2020), but its influence in our field is palpable. This manifests in several diverse ways within medicine and medical education. For example, conversations and initiatives to tackle the gender pay gap, and to ensure equal representation of women in senior academic and clinical positions, are rooted in the ideals of freedom for women and the right to political equality (Sharma, 2019). Much gender bias research is rooted in liberal feminist ideals e.g., discussion of the ‘glass ceiling’, ‘sticky floor’, or ‘labyrinth’ in relation to women’s career trajectories (as we have discussed earlier in this chapter) all imply that women are somehow trapped, and that the ultimate aim to feminist activism should be freedom from inequality in the workplace.

#### Example

One example of a popular theory rooted in liberal feminism is the suffragette Wollstonecraft’s personhood theory, put forth in her feminist critique ‘A Vindication of the Rights of Women’ (1796), in which she argues that inequality between men and women exists due to differences in their educations, rather than a popular explanation for inequality at the time- that women are naturally inferior to men. Wollstonecraft puts forth that men and women should both be treated as rational beings, and imagines a new social order founded on reason, in which women are educated alongside men, in a way that was commensurate with their social status. Wollstonecraft (1796) stops short of declaring men and women equal and is careful to note ‘let it not be concluded that I wish to invert the order of things; I have already granted, that, from the constitution of their bodied, men seem to be designed… to attain a greater degree of virtue’. This has since subjected Wollstonecraft, and liberal feminism in a wider sense, to the critique that this approach only seeks to make changes within existing structures (e.g., existing social orders or political systems), rather than upend, abolish or radically change those structures themselves (GWAnet Central Asia, 2021).

#### Critique

Though liberal feminism has attracted much mainstream attention (so much so, it is sometimes referred to as ‘mainstream feminism’) (Maynard, 1995) it has been subject to further significant critiques on the basis of the way in which it neglects to consider the impact of intersecting factors that may oppress one’s freedom such as ethnicity, sexuality and class. Others still argue that the way in which liberal feminism focuses on the freedom of individuals means that it is unable to sufficiently conceptualise injustice that occurs to groups of people (Yuracko, 2003). Though liberal feminism provided an important foundation for several feminist movements, given increasing recognition that gender bias and oppression cannot be conceptualised from a narrow, unidimensional viewpoint (Connelly and Barriteau, 2000), considering approaches to feminist theories which counteract some of the critiques levied against liberal feminism is important and worthwhile.

### 2nd wave: Existentialist feminism

#### Description

*One is not born, but becomes, a woman*

## Simone de Beauvoir, The Second Sex

Perhaps one of the existentialist philosopher Simone de Beauvoir’s most famous quotes is the opening line to the second section of her book ‘The Second Sex’ (1953) – ‘One is not born, but becomes, a woman’. Simply put, de Beauvoir advances the position that biology is not what makes a woman – life experiences are, and women learn their role as a woman from tacit messaging and men. Existentialism is a philosophy which proposes that existence proceeds essence- we are not born women, we become women. De Beauvoir’s existential approach to feminism was influential in spurring a move into the second wave of feminism, which was more concerned with the experience of women than the suffrage-concerned first wave of feminism had been. Second wave feminism was originally referred to as women’s liberation (Thompson, 2001).

An existential approach to feminism emphasises freedom, as does liberal feminism, but also interpersonal relationships and the experience of living as a woman. Moving away from a primary focus on the individual, existentialist feminism explores and attempts to exposure the social structures and conventions that define, impose and reinforce gender roles so that women can make considered, free choices about their lives and the way in which they wish to live.

### Example

One example of an existential feminist theory is norm theory, when it is applied to matters of gender. In ‘norm theory’, men are considered typical members of the ‘human’ category, whilst women are deviant (Hibbs, 2014). Norm theory includes de Beauvoir’s feminist philosophy that men are treated as the standard- the norm- within society, and women as ‘the Other’. The Second Sex (1953) includes reference to this theory- ‘He is the Subject, he is the Absolute – she is the Other’. The concept of women as ‘the Other’ and the proliferation of norm theory has also since become a critical concept in a host of other theories, including theories that analyse the oppression of colonised, enslaved and other exploited peoples (Epstein, 2014).

Within health professions education, an explicitly named existentialist approach to feminist theory seems to have been adopted much less frequently than a liberal approach (indeed, existentialist feminism is not an approach named within Sharma’s typology (2019) and upon review of the literature we could see little theory clearly utilised within this approach). This is despite evidence that the othering of women still occurs within medical practice, research and education (Verdonk et al., 2009; Risberg et al., 2009). We see opportunities for existentialist investigation within several topics of contemporary interest within medical education, especially research questions and educational structures which concern power and sociocultural norms. Recently, in the wake of disruptions as a result of COVID-19, evidence has emerged demonstrating that women have been disproportionally affected by the pandemic in regard to their career and economic status (Durygin, 2020). With school closures, the reduced availability of childcare and potential of unwell relatives, women have been expected to cease working to look after children or family members. Women have also been expected to assume additional housekeeping responsibilities during the pandemic, and often assumed the additional responsibility of supporting their male partners to work at home, without due credit. De Beauvoir (1953) notes that ‘economically, men and women almost form two castes’- recent evidence suggests COVID-19 may have widened inequalities between men and women within society as a result of shifting social structures and conventions (Madgavkar et al., 2020). Women’s role as ‘the Other’ has been affirmed- men are the norm, and so prioritised, and gender roles within the home reinforced during the pandemic. This is one way in which we envision an existentialist approach to feminism may help cast light on the experiences of women within our field during COVID-19, and the sad but likely possibility of economic and career differences between men and women at similar stages in their careers as a result of COVID-19.

#### Critique

Some scholars have outlined critiques of existentialist approaches to feminism that it is important we consider. Critiques exist of the approach more broadly, and of de Beauvoir’s work specifically. Given the foundational role of de Beauvoir within this approach to feminism, and the way in which we have discussed her theory here, we will consider critiques specifically as they pertain to her work. Some of criticised de Beauvoir’s seminal text ‘The Second Sex’ as only speaking to other white, middle class women, and neglecting to consider the experiences of less socioeconomically privileged groups (Silverio, 2019). Others have noted an inconsistency between de Beauvoir’s works, highlighting that the women in her fictional works often conform to norms, rather than fighting against the Otherness they are oppressed with (Dolske, 2014). Perhaps most significantly for a field concerned with practicality, de Beauvoir has been critiqued for her inability to transfer her existentialist feminist philosophy into praxis (Silverio, 2019), which could cast doubt on the use of this approach to affect meaningful change within health professions education. Though existentialist feminism goes further than liberal feminism to centre and highlight the experiences of women, critiques of the approach are not insignificant, and approaches to feminism originating within later waves also warrant consideration.

#### 3rd wave: Queer feminism

##### Description

Within its third wave, feminism experienced a ‘queer turn’ (Berger, 2013), where feminism and queer theory met to establish queer feminism as an approach to feminist issues. Unfortunately, the term queer is often misunderstood within medicine, but it can be defined as ‘an umbrella term used to describe individuals who are from sexual or gender minority groups’, typically describing those who do not identify as heterosexual or cisgender (Finn et al., 2021). Queer theory is a field within critical theory that studies, critiques and challenges dominant social norms, especially those relating to sexuality (Jackson, 2021).

Though feminism and queer theory have been historically been placed into opposition with one another, with some scholars suggesting feminism’s focus should be gender, whilst queer theory’s focus should be sexuality (Butler, 1994), the approaches are increasingly interconnected as both feminism and queer theory focus on the ‘construction and deconstruction of the naturalised and binary categories of gender and sexuality’ (Liljeström, 2019). Contemporarily, queer feminism pays attention to sexuality, gender, and their plasticity (Berger, 2013). Queer feminism expands the definition of feminism to explicitly include queer people, with proponents of the approach critiquing first and second wave feminism for focusing only on equality between men and women, excluding non-binary genders, and ignoring the ways in which patriarchal issues can harm people of any gender (Mann and Huffman, 2005; Coleman, 2009).

#### Example

One notable example of a queer feminist theory is Judith Butler’s theory of gender performativity. Building on the work of de Beauvoir within The Second Sex, Butler (1988) notes a difference ‘between sex, as a biological facticity, and gender, as the cultural interpretation or signification of that facticity’. Given that gender involves cultural interpretation, Butler argues that gender should be conceptualised as performative. Within this context, use of the term ‘performative’ implies that individuals construct and perform gender to a social audience. Butler (1988) further suggests that performances of gender are shaped by historical social norms and practices, transmitted between individuals and generations in a way that reinforce a gender binary, and legitimatises the idea that gender is natural and innate, between men and women.

Though Butler’s theory regarding the performativity of gender could be used within health professions education to explore the acts that performatively constitute a wide range of social practices, we could not see within our literature search that Butler’s theory has been used within our field. Later in this paper, we will explore the ways in which queer feminist approaches to teaching may better inform health professions education. In regard to research, we see relevance of queer approaches to feminism, and Butler’s theory of performativity to many potentially fruitful areas of inquiry, including: the exploration of students’ and trainees’ gendered experiences within education; the gendered experiences of faculty and those in leadership positions; and exploration of professional identity development which conceptualises identity as performative, and considers the impact of gender.

#### Critique

As with all theoretical approaches, queer feminism has been subject to critique by some scholars. Some of these critiques are associated with queer feminism’s origins in queer theory. Feldman (2009) argues that queer theory erases bisexuality, and that a masculine bias exists within the field that stems from the way in queer theory relies on Foucauldian theories of sexuality. There are proponents of the view that queer theory in practice is less inclusive than definitions within the field suggest- Jeffreys (1994) argues that where the term queer is used, it is most often used to mean ‘white gay male’, so that whilst claiming to be ‘new and uniquely liberating’, queer theory and, by extension, queer feminism, erases the experiences of many feminists, lesbians, bisexuals and people of colour. There have been responses within the queer feminist approach to these critiques, such as the establishment of queer of colour theory by Ferguson (2004), an approach which places more emphasis on intersections of race, gender, sexuality and class. Yet, there are critiques still of this response- Moore (2011) puts forth the view that queer theory cannot ‘generate and sustain analysis and action that aggressively counters… white racist ideology, and white privilege’, calling for approaches which ‘interrogate and deconstruct notions of whiteness and white privilege’ more explicitly. Intersectional feminism is an alternative approach to feminism which foregrounds considerations of racial oppression.

#### 4th wave: Intersectional feminism

##### Description

Intersectional feminism is an approach to feminism used to theorise, scrutinise, and challenge identity and oppression – it is concerned with power, and how this manifests between individuals, but also within structures (Nash, 2008). The term was coined by Crenshaw (1989) and has since become the most common way of ‘conceptualising the relation between systems of oppression which construct or multiple identities and our social locations in hierarchies of power and privilege’ (Carastathis, 2014). Intersectional feminism can be seen as a response to the critique that feminist approaches in previous waves only consider the rights and experience of middle class, white women (Crenshaw, 1989).

Monrouxe (2015) defines intersectional medical education research as research which aims to understand the impact and interaction between multiple discriminations on individuals’ senses of self and experiences. What is key to intersectional approaches is that social categories such as gender, race, ethnicity, class, and sexuality are perceived as inseparable – they cannot be studied or considered in isolation, as these categories connect to influence individuals’ lived experiences and identities (Monrouxe, 2015).

Intersectional feminism comprises a group of theories united by this conceptualisation. Collins (1990) theorises an interlocking matrix of oppression where intersections of social inequality contribute to the oppression of individuals. This matrix can be used to explain complex issues of oppression in regard to race, class, age, sex and gender, where these concepts are conceptualised as interconnected (Patricia Hill Collins: Intersecting Oppressions, 2005). Within the matrix of oppression, there are many different ways in which a person may be challenged or oppressed. The matrix also notes the role and impact of privilege, considering an individual’s interactions and experiences based on different interconnected social inequalities and privileges (Johnson, 2005). Whilst a person may be privileged in one area of the matrix (for example, they may be middle class or male), they can still be oppressed in regard to other aspects of their identity (for example, they may experience racial discrimination as a person of colour). (Wyatt et al., 2020b; Wyatt et al., 2020a)

#### Example

Within health professions education, recent research reveals the pressing need for intersectional approaches to teaching, research and scholarly activity. Take for example issues such as the migration of healthcare professionals, health inequalities, global classrooms and global health curricula, all of which have gained more attention in recent years, especially in light of the COVID-19 pandemic. The pandemic has evidenced the inequity in health outcomes. While health professionals, particularly those in developed countries, are now expected to be proficient in the management of global diseases and newly emerging infections, there is an expectation that this must be coupled with cultural sensitivity to global migration and ethnic minority populations (Kapilashrami and Hankivsky, 2018). What is often absent is awareness of intersectionality, and the way multiple oppressions may be experienced (Smith, 2013) by those they are studying with, working with, teaching, or treating, perhaps the result of a curricula gap.

Intersectionality offers a lens through which to bring (1) increased awareness of significant differences within populations that are often presented as homogenous, (2) consider multifaceted power structures and processes, and (3) interactions between multiple research sites. The insights afforded by intersectional analyses are essential in researching and explaining intra and inter-group differences, charting new policies or developing global curricula, as well as exploring how oppression and power manifests in these contexts for different stakeholders and their intersecting identities and positions.

If we further consider the healthcare workforce, professional identity research is one area in which this conversation has recently mushroomed. Wyatt et al. (2020a; 2020b; 2021) note that race, ethnicity and ‘sociohistorical context’ are not prominent considerations within medical education professional identity research or literature (Cruess et al., 2014), highlighting that this has resulted in an underappreciation of minoritized physicians’ experiences, and the use of identity theory which has not been validated for use within marginalised communities. The multiple, intersecting identities a studied population holds are often forgotten or trivialised, thus power, oppression and heterogeneity are ignored. Tsouroufli et al. (2011) highlighted the need for more intersectional approaches to medical education research ten years ago, a call which is still yet to receive the attention it deserves. The matrix of oppression has not yet received attention as a theory within health professions education or research, though it has made a notable impact in adjacent fields, where it has been used to illuminate the interconnected patterns of privilege and marginalisation in regard to race, class, gender, age etc. within social institutions and communities (Winker and Degele, 2011). We hypothesise that Collins’ theory may offer one theoretical lens through which health professions education scholars can more appropriately and considerately explore the experiences of minoritized physicians, going some way to answer the call of Tsouroufli and build on the critically important research conducted by Wyatt and colleagues.

#### Critique

As with all the feminist approaches we have outlined thusly, there are critiques associated with intersectional feminism it is important to understand. Intersectional feminism relies heavily on feminist standpoint theory, which has its own associated critiques (Reilly-Cooper, 2013). Feminist standpoint theory maintains that oppressed people are best placed to judge their own experiences of oppression, yet this creates a paradox when people who are similarly oppressed describe different experiences. This can make deciding on common actions to address oppression difficult (Reilly-Cooper, 2013). Critiques also exist in regard to the way in which intersectionality denies Jewish people’s experiences of oppression, leading to critiques that the approach is anti-Semitic (Schwartz, 2020). These are difficult critiques to respond to and must be meaningfully considered by those choosing to utilise intersectional approaches.

## The challenges to increasing the use of feminist theory within health professions education

Within this paper, we have advanced our argument for increasing the use of feminist theories to inform research and education within our field by demonstrating their possible value. Yet, though feminism is by no means a new school of thought, uptake of feminist theories within health professions education has been slow, especially by comparison to the popularity and use of such theories within adjacent fields such as sociology. It is important to briefly consider the challenges educators and scholars within health professions education may face in regard to the use of feminist theory, both to raise awareness of these barriers and to consider ways in which they may be surmounted.

Though the demand for theory within health professions educational settings and research is increasingly, there is a reluctance, for some, to engage with theory (including feminist theory). Theory is perceived as nebulous, troublesome and of little practical use (Kaufman, 2003; Teunissen, 2010; Albert et al., 2007). Reports exist that those new to medical education (including graduate students) find theory difficult to engage with (Laksov et al., 2017). Where theory is used, theory created within the field of health professions education itself is often preferred to the exploration of sociological or psychological theories more broadly (Albert et al., 2020). As a field, it is important we formally train blossoming educators and researchers in the appropriate use of educational and sociological theories, including feminist theory use. Encountering theory in a supportive, educational space may offer scholars within our field increased opportunities to explore the importance and use of theory, countering future reluctance to engage. The practicality of theory should also be highlighted – psychologist Kurt Lewin’s old adage ‘there is nothing so practical as a good theory’ rings true here. Theories are what make abstract experiences or conditions in the world intelligible or understandable (Varpio et al., 2020b). This is crucial to our work as educators and researchers, for without appropriate understanding of what is happening around us, how can we decide the most appropriate course of action to resolve the issues we identify? Health professions education scholars should be encouraged to seek and use theories from outwith the field – there are a wealth of theories that exist beyond our field’s boundaries, and early career educators and researchers must be appropriately equipped with the skills to identify and apply a wide range of theories, advancing the interdisciplinary nature of our field. Introducing feminist domains to theories already popular or created within our field should also be highlighted as an interesting direction for research – such as Samuriwo et al.’s (2020) research into medical students’ perceptions of gender, which introduces the concept of ‘gendered communities of practice’ to explain the culture students experience in clinical practice whereby their gender influences the teaching and learning they receive. We anticipate similar value could be inferred by considering theories such as transformative learning, experiential learning theory, behaviourism learning theory, sociocultural theory, and humanistic theory through feminist lenses. This is only a small glimpse into the ways in which feminist theory may be useful when it comes to theory use in our field. We encourage readers to consider the theories they are most familiar with and consider whether depth or value may be added through feminist exploration of these theories, in particular through an intersectional lens. We also note Paton et al’s (2020) recommendations to explore absence research in order to unearth potential in the theoretical and methodological approaches within our field. As they suggest, absence research could shed light on aspects of health professions education that have received limited attention thus far.

There is a metaphorical elephant in the room regarding the use of feminist theory, and this concerns the stigma associated with the feminist label. Despite decades of progress, there is what Edley and Wetherell (2001) term a ‘Jekyll and Hyde’ quality to the perceptions of feminists, where they are admired widely by some, but criticised and stigmatised by others. A pervasive negative, ‘Mr Hyde’ image of feminism exists, where women are lauded as militant, ‘Femi-Nazi’, man-hating, difficult, angry, ‘bra-burning crazies’(McCabe, 2005; Swirsky and Angelone, 2014; Remenyi, 2016). It is hoped that we don’t have to highlight that these stereotypes are nonsense. Yet, this stigma exists and can cause fear amongst all genders at being associated with feminist education, research, or causes (Arcieri, 2017). Indeed, we have personally seen passionate educators and scholars shrink away from tackling issues perceived as feminist, in the fear that highlighting such issues within institutions would subject them to repercussions. This reveals an unacceptable culture within some health professions institutions that seeks to silence the advocacy of feminism by propagating vicious stigma against those who associate with feminist labels or causes. Though women are increasingly well-represented within medicine, they are poorly represented in senior health professions education management and leadership roles, hindered by a lack of institutional policy, specific requirements in regard to research productivity or credentials, and the way in which women are forced to navigate more complex social structures whilst working towards promotion (Varpio et al., 2020a; Marsh and Chod, 2017; Fitzpatrick, 2012). An improved awareness of the gendered experiences of women working within health professions education is necessary to inform policy which aims to ensure equal representation of genders within senior roles (Alwazzan and Al-Angari, 2020). Increased representation of all genders within senior institutional roles may go some way to combating the cultural stigma associated with feminist education, research and activism within health education institutions.

As we have showcased throughout this paper, there are a multitude of feminist approaches and theories. It can be difficult, as an early career researcher, or a feminist theory novice, to know where to begin in regard to learning more about feminist theory and its use. Within Table 1 we have supplied a sample of feminist theory references we hope may act as a springboard to engagement in feminist theory literature. These have been selected on the basis of relevance to the field and our lived experiences. In addition to becoming familiar with feminist theory literature, we recommend you seek the advice of a colleague experienced in theory use or knowledgeable of feminism for support. Reviewing empirical studies published within health professions education journals may help you see how feminist theories can be used and applied in practice, whilst potentially opening your eyes to new feminist theories. Long-term, building a network of health professions education scholars interested in feminist theory may help add depth to your work and will provide a space for scholarly discourse. Professional networks can be difficult to establish – don’t be afraid to reach out to scholars using the theories you use or admire in the field, join special interest groups, or interact with scholars online through social networking sites such as Twitter.

**Table 1:** Foundational feminist texts and overview references

Reference	Brief description
Feminism is for Everybody, bell hooks	Written from a radical feminist position, bell hooks positions the learning of feminist theory and history as core parts of the practice of freedom. A thought-provoking overview for readers new to interdisciplinary feminism. Contests the view that feminism is no longer necessary.
The Second Sex, Simone de Beauvoir	This book offers a historical overview of women’s disadvantaged position in society, putting forth an existentialist feminist agenda. Considers gender ‘facts and myths’ that de Beauvoir attempts to deconstruct, and a section on ‘Lived Experience’, where the ways in which women experience sexism are considered.
We Should All Be Feminists, Chimamanda Ngozi Adichie	Argues that ‘feminist’ is not an insult, but a label that should be embraced by all. The central premise of the book is that being a feminist means understanding and acknowledging that sexism exists. Concludes on the note that people harm both men and women by teaching them to adhere to strict gender roles.
Gender Trouble, Judith Butler	In this book, Butler argues that gender is an improvised performance. Foundational to queer theory and queer feminism. Questions the basis that supposedly fixed identities such as ‘masculine’ and ‘feminine’ have and argues for a move away from such binaries.
The Oxford Handbook of Feminist Theory, Lisa Disch and Mary Hawkesworth	Offers a comprehensive overview of popular approaches to feminism, demonstrating the interdisciplinary nature of feminist theory that cannot be bounded by traditional field limits. Argues that feminist theory is crucial to understanding and illuminating power dynamics and inequalities which operate within contemporary society.
Feminist Theory: A Philosophical Anthology, Ann Cudd and Robin Andreasen	Centres seven philosophical questions regarding feminism, the concepts of sex and gender, and women’s experiences. Offers an introductory overview to feminism as a philosophical movement. Considers topics such as the nature of sexist oppression, sex/gender distinctions, feminist ethics, and what constitutes freedom.
Black Feminist Anthropology: Theory, Politics, Praxis and Poetics, Irma McClaurin and Johnetta Betsch Cole	A collection of essays from nine Black feminist anthropologists. Focuses on the experiences of the authors, and how this has influenced their practice as anthropologists and ethnographers.
Intersections between Feminist and Queer Theory, Diane Richardson, Janice McLaughlin, Mark Casey	Explores debates between feminist and queer theory to highlight connections and identify new directions for queer feminist research.
Contemporary Feminist Research: From Theory to Practice, Patricia Leavy and Anne Harris	A book that covers the breadth of feminist theory within research. The text offers practical strategies, methods, and well explained theories. Case studies help cement concepts, and extensive reading lists provided,
Demarginalizing the Intersection of Race and Sex: A Black Feminist Critique of Antidiscrimination Doctrine, Feminist Theory and Antiracist Politics, Kimberle Crenshaw	The work of Kimberle Crenshaw, who coins the term ‘intersectionality’ in this insightful essay. Uses analogies to demonstrate the meaning of the concept. Highlights the absence of Black women from analyses of gender oppression, and the need for an intersectional approach to fight sexism in a racist society.

**Fig. 3:**
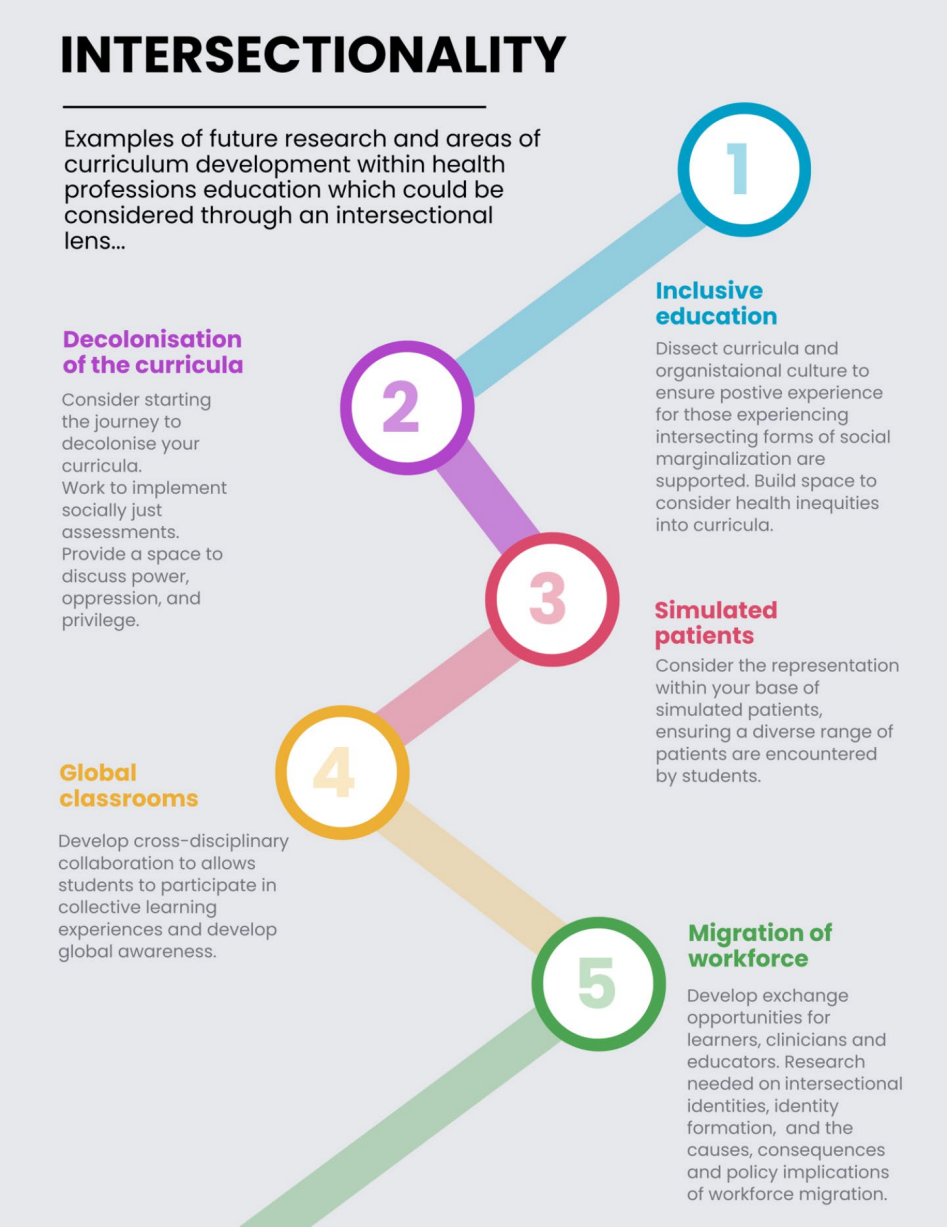
Examples of future research and areas of curriculum development within health professions education which could be considered through an intersectional lens

Finally, in order to demonstrate the potential to integrate feminist theory approaches into these health professions education, we provide Fig. 3. In this figure we have delineated how the theory of intersectionality could be explored within future research and areas of curriculum development.

## Concluding remarks

In this theoretical commentary, we have discussed the heterogenous ways in which feminism has been mobilised for education and research both within, and outwith, the field of health professions education. Though the post-feminist movement may decree ‘ding dong, feminism is dead!’, feminism is just as necessary as ever and lives on in a diverse range of approaches that have generated a multifaceted spectrum of feminist theories.

Yet, feminist approaches have not received the attention they deserve within health professions education and have been underutilised in the exploration of gendered experiences and inequalities. As the fourth wave of feminism surges forwards, approaches within health professions education must keep pace- gender should not be considered or studied in isolation. Though this need is recognised within health professions education, it is less clear what this means in practice, and further guidance is necessary from experienced intersectional scholars as to how intersectionality can meaningfully influence health professions research questions, projects, and teaching. Though conversations have turned to what intersectionality means within the context of health professions education research (Monrouxe, 2015), we must not forget our duty to students and also imbue our approaches to teaching and learning with intersectionality. bell hooks (2010) highlights that feminist approaches to teaching and learning stress ‘the primacy of critical thinking, of linking education and social justice’- this should be a central aim of healthcare teaching in its endeavour to produce socially conscious advocates.

Ongoing feminist explorations and critiques are essential to reducing the health inequalities that exist between genders and are amplified by intersecting vectors of oppression. Though reluctance concerning the use of theory, and stigma associated with the feminist label exist, as bell hooks puts forth- ‘feminism is for everybody’. Perhaps it is time to reclaim one of the terms associated with gender bias within medicine and subvert it to call for an active movement within health professions education to promote feminist agendas and move towards hook’s steer. There is power in reclaiming words intended as insults or those that carry negative meanings for women- the term ‘suffragette’ was incepted as a demeaning way to refer to women invested in liberation activities in the early 20th century yet is now associated with female empowerment and emancipation (Montell, 2019). To subvert a term used to refer to the discrimination experienced by women when they are bypassed for an opportunity on the basis of their gender, we ‘ova-look’ feminist theory within health professions education at our own peril. We call for all health professions education scholars who have previously ova-looked feminist theory to start considering how this rich, theoretical approach can meaningfully contribute to the plight for gender equity within healthcare and healthcare education.

## Footnotes

1 Protected characteristics are specific aspects of a person’s identity defined by the Equality Act 2010. The ‘protection’ relates to protection from discrimination. They are also known as protected classes or prohibited grounds.

## References

[CR1] Albert M, Hodges B, Regehr G (2007). Research in medical education: balancing service and science. Advances in Health Sciences Education.

[CR2] Albert M, Rowland P, Friesen F, Laberge S (2020). Interdisciplinarity in medical education research: myth and reality. Advances in Health Sciences Education.

[CR3] Alwazzan L, Al-Angari S (2020). Women’s leadership in academic medicine: a systematic review of extent, condition and interventions. BMJ open.

[CR4] Arcieri, A. (2017). The stigma of the feminist label and its reduction. Thesis, The University of Sydney

[CR5] Artwińska, A., & Mrozik, A. (Eds.). (2020). *Gender, Generations, and Communism in Central and Eastern Europe and Beyond*. Routledge

[CR7] Berger A (2013). The Queer Turn in Feminism: Identities, Sexualities, and the Theater of Gender.

[CR8] Blackman, M. (2021). Malorie Blackman Quotes. https://quotesia.com/malorie-blackman-quotes Accessed 11 November 2021

[CR10] Brown, M.E.L., Hunt, G.E.G., Hughes, F., & Finn, G.M. (2020). ‘Too male, too pale, too stale’: a qualitative exploration of student experiences of gender bias within medical education. BMJ Open, 10 (8):e039092. doi: 10.1136/bmjopen-2020-039092 10.1136/bmjopen-2020-039092 PMC7430333 32792453

[CR11] Butler J (1988). "Performative Acts and Gender Constitution: An Essay in Phenomenology and Feminist Theory". Theatre Journal.

[CR12] Butler J, Weed E, Schor N (1994). “Against Proper Objects”. Feminism Meets Queer Theory.

[CR13] Carastathis A (2014). The concept of intersectionality in feminist theory. Philosophy Compass.

[CR14] Choo E, DeMayo R (2018). A lexicon for gender bias in academia and medicine. BMJ.

[CR16] Coleman J (2009). An introduction to feminisms in a postfeminist age. Women’s Studies Journal.

[CR17] Collins P, Collins P (1990). Black feminist thought in the matrix of domination. Black feminist thought: Knowledge, consciousness, and the politics of empowerment.

[CR18] Connelly J, Barriteau P (2000). Theoretical perspectives on gender and development.

[CR19] Crenshaw K (1989). Demarginalizing the Intersection of Race and Sex: A Black Feminist Critique of Antidiscrimination Doctrine, Feminist Theory and Antiracist Politics.

[CR20] Cruess R, Cruess S, Boudreau J, Snell L, Steinert Y (2014). Reframing medical education to support professional identity formation. Academic Medicine.

[CR22] De Beauvoir S (1953). The second sex.

[CR23] Dolske G (2014). Existential Destruction: de Beauvoir’s Fictional Portrayal of Woman’s Situation. Women’s Studies.

[CR24] Durygin, M. (2020). *Simone de Beauvoir and a period of transition* (pp. 1–7). Prospects 10.1007/s11125-020-09513-x PMC7556556 33078032

[CR25] Edley N, Wetherell M (2001). Jekyll and Hyde: Men’s constructions of feminism and feminists. Feminism and Psychology.

[CR26] Epstein C (2014). The postcolonial perspective: an introduction. International Theory.

[CR27] Ewing-Nelson, C. (2020). *Part-time workers are paid less*. have less access to benefits – and most are women. National Women’s Law Center. Retrieved May 3 (2021). from: https://nwlc-ciw49tixgw5lbab.stackpathdns.com/wp-content/uploads/2020/02/Part-Time-Workers-Factsheet-2.26.20.pdf

[CR29] Feldman S (2009). Reclaiming sexual difference: What queer theory can’t tell us about sexuality. Journal of Bisexuality.

[CR30] Ferguson R (2004). Aberrations in Black: Toward a queer of color critique.

[CR31] Finn G, Ballard W, Politis M, Brown M (2021). It’s not alphabet soup- supporting the inclusion of inclusive queer curricula in medical education. British Student Doctor Journal.

[CR32] Finn G, Morgan J (2020). ’From the sticky floor to the glass ceiling and everything in between: A systematic review and qualitative study focusing on gender inequalities in Clinical Academic careers’.

[CR33] Finn, G. M., Quinton, H., & Hafferty, F. W. (2022). The Significance of the Body in Health Professions Education. In: M. E. Brown, M. Veen, & G. M. Finn (Eds.), A Journey Towards Mutual Understanding: Applied Philosophy for Health Professions Education. Springer Publishing House, USA, in press

[CR34] Fitzpatrick S (2012). A survey of staffing levels of medical clinical academics in UK medical schools as at 31 July 2011.

[CR35] Fortier N (2020). COVID-19, gender inequality, and the responsibility of the state. International Journal of Wellbeing.

[CR36] Froldi G, Dorigo P (2020). Endothelial dysfunction in Coronavirus disease 2019 (COVID-19): Gender and age influences. Medical hypotheses.

[CR6] GWAnet Central Asia. (2021). ‘History and theory of feminism’. Retrieved May 3, 2021, from: http://www.gender.cawater-info.net/knowledge_base/rubricator/feminism_e.htm

[CR37] Hibbs, C. (2014). Androcentrism. Encyclopedia of Critical Psychology, 2–24

[CR38] hooks (2000). *Feminism is for everybody: passionate politics*. London:Pluto Press

[CR39] hooks, b. Teaching critical thinking: practical wisdom. (New York:Routledge)

[CR40] Humm M (2015). A readers guide to contemporary feminist literary criticism.

[CR41] Jackson, J. (2021). Queer theory: Resources. Retrieved May 3 2021, from: https://researchguides.uic.edu/queertheory

[CR42] Jeffreys S (1994). The queer disappearance of lesbian sexuality in the academy. Women’s Studies International Forum.

[CR43] Johnson A, Ritzer G (2005). Matrix of domination. (In. Encyclopedia of social theory.

[CR44] Kapilashrami A, Hankivsky O (2018). Intersectionality and why it matters to global health. The Lancet.

[CR45] Kark R, Eagly A, Chrisler JC (2010). Gender and leadership: Negotiating the labyrinth. (In. Handbook of gender research in psychology.

[CR46] Kaufman D (2003). Applying educational theory in practice. BMJ.

[CR47] Laksov K, Dornan T, Teunissen P (2017). Making theory explicit-An analysis of how medical education research (ers) describe how they connect to theory. BMC Medical Education.

[CR48] Laughey W, Sangvik Grandal N, Finn G (2018). Medical communication: the views of simulated patients. Medical Education.

[CR49] Lazarus, M., & Sanchez, A. (2021). Redefining anatomical language in healthcare to create safer spaces for all genders. https://lens.monash.edu/@medicine-health/2021/05/17/1383207/redefining-anatomical-language-in-healthcare-to-create-safer-spaces-for-all-genders. Accessed: 01 June 2021

[CR50] Leavy P, Harris A (2018). Contemporary feminist research from theory to practice.

[CR51] Liljeström M (2019). Feminism and queer temporal complexities. SQS–Suomen Queer-tutkimuksen Seuran lehti.

[CR52] Madgavkar, A., White, O., Krishnan, M., Mahajan, D., & Azcue, X. (2020). COVID-19 and gender equality: Countering the regressive effects. McKinsey Global Institute. Retrieved 3 May, 2021, from: www. mckinsey. com/featured-insights/future-of-work/covid-19-and-gender-equality-countering-the-regressive-effects

[CR53] Maynard M (1995). "Beyond the ‘big three’: the development of feminist theory into the 1990s". Women’s History Review.

[CR54] Mann S, Huffman D (2005). The decentering of second wave feminism and the rise of the third wave. Science & society.

[CR55] Marsh J, Chod R (2017). Recruiting Faculty Leaders at US Medical Schools: A Process Without Improvement?. Academic Medicine.

[CR56] McCabe J (2005). What’s in a label? The relationship between feminist self-identification and “feminist” attitudes among U.S. women and men. Gender and Society.

[CR57] Miller V, Flynn P, Lindor K (2012). Evaluating sex and gender competencies in the medical curriculum: a case study. Gender medicine.

[CR59] Monrouxe L (2015). When I say… intersectionality in medical education research. Medical Education.

[CR60] Montell A (2019). Wordslut: A Feminist Guide to Taking Back the English Language.

[CR61] Moore D (2011). An interrogation of the black presence in the queer project. Trans-Scripts.

[CR62] Nash J (2008). Re-thinking intersectionality. Feminist review.

[CR63] Nicholson, L. (2010). ‘Feminism in ‘Waves’: Useful Metaphor or Not?‘. New Politics. Retrieved May 3, 2021, from: https://newpol.org/issue_post/feminism-waves-useful-metaphor-or-not/#:~:text=It%20was%20useful%20because%20it,a%20long%20tradition%20of%20activism

[CR64] Ovseiko P, Chapple A, Edmunds L, Ziebland S (2017). Advancing gender equality through the Athena SWAN Charter for Women in Science: an exploratory study of women’s and men’s perceptions. Health research policy and systems.

[CR65] Paton M, Naidu T, Wyatt TR, Oni O, Lorello GR, Najeeb U, Kuper A (2020). Dismantling the master’s house: new ways of knowing for equity and social justice in health professions education. Advances in Health Sciences Education.

[CR66] Patricia Hill Collins: Intersecting oppressions. (n.d.). Retrieved May 3 (2021). from: http://uk.sagepub.com/sites/default/files/upm-binaries/13299_Chapter_16_Web_Byte_Patricia_Hill_Collins.pdf

[CR67] Reilly-Cooper, R. (2013). “Intersectionality and identity politics”. More Radical With Age. Retrieved May 1, 2021, from: https://rebeccarc.com/2013/04/15/intersectionality-and-identity-politics/

[CR68] Remenyi, K. (2016). *“I’m not a feminist, but… An investigation into the attitudes of University of Kent students who identify with the objectives of the feminist movement, but do not identify as a feminist* (pp. 1–12). E-International Relations

[CR69] Risberg G, Johansson E, Hamberg K (2009). A theoretical model for analysing gender bias in medicine. International journal for equity in health.

[CR70] Royal College of Obstetrics and Gynaecologists (2019). Better for Women: Improving the health and wellbeing of girls and women.

[CR71] Samuriwo R, Patel Y, Webb K, Bullock A (2020). ‘Man up’: Medical students’ perceptions of gender and learning in clinical practice: A qualitative study. Medical Education.

[CR72] Schwartz, G. (2020). *“Beyond Skin-Deep: The Exclusion of the Jews from Intersectional Discourse”*. The Stanford Review

[CR73] Sen G, Östlin P (2008). Gender inequity in health: why it exists and how we can change it. Global public health.

[CR74] Sharma M (2019). Applying feminist theory to medical education. The Lancet.

[CR75] Silverio S (2019). A critical review of how existentialism and its men influenced the feminism of Simone de Beauvoir. British Mensa’s: Androgyny.

[CR76] Smelser N, Baltes P (2001). International encyclopedia of the social & behavioral sciences.

[CR77] Smith S (2013). Black feminism and intersectionality. International Socialist Review.

[CR78] Swirsky J, Angelone D (2014). Femi-nazis and bra burning crazies: A qualitative evaluation of contemporary beliefs about feminism. Current Psychology.

[CR79] Tesch B, Wood H, Helwig A, Nattinger A (1995). Promotion of women physicians in academic medicine: glass ceiling or sticky floor?. Jama.

[CR80] Teunissen P (2010). On the transfer of theory to the practice of research and education. Medical Education.

[CR81] Thompson D (2001). Radical feminism today.

[CR82] Tsouroufli M, Rees C, Monrouxe L, Sundaram V (2011). Gender, identities and intersectionality in medical education research. Medical Education.

[CR83] Varpio L, Harvey E, Jaarsma D, Dudek N, Hay M (2020). Attaining full professor: Women’s and men’s experiences in medical education. Medical Education.

[CR84] Varpio L, Paradis E, Uijtdehaage S, Young M (2020). The Distinctions Between Theory, Theoretical Framework, and Conceptual Framework. Academic Medicine.

[CR85] Verdonk P, Benschop Y, De Haes H, Lagro-Janssen T (2009). From gender bias to gender awareness in medical education. Advances in Health Sciences Education.

[CR86] Voet, M., & Voet, R. (1998). *Feminism and citizenship*. Sage. p25

[CR87] Whitehead C, Kuper A, Webster F (2012). The conceit of curriculum. Medical education.

[CR88] Williams, J. (2005). The glass ceiling and the maternal wall in academia. New Directions for Higher Education, 130, 91–105

[CR90] Williams J, Dempsey R (2014). What Works for Women at Work: Four Patterns Working Women Need to Know.

[CR89] Williams, J., Alon, T., & Bornstein, S. (2006). Beyond the ‘chilly climate’: Eliminating bias against women.Thought & Action.,79

[CR91] Winker G, Degele N (2011). Intersectionality as multi-level analysis: Dealing with social inequality. European Journal of Women’s Studies.

[CR92] Wollstonecraft M (1796). A Vindication of the Rights of Woman.

[CR93] World Health Organisation. (2021). (2020) ‘Where do we stand on women’s health in 2020?‘. World Health Organisation, Regional Office for Europe. Retrieved May 3, 2021, from: https://www.euro.who.int/en/health-topics/health-determinants/gender/news/news/2020/3/where-do-we-stand-on-womens-health-in-2020

[CR94] Wyatt T, Balmer D, Rockich-Winston N, Chow C, Richards J (2021). ‘Whispers and shadows’: A critical review of the professional identity literature with respect to minority physicians. Medical Education.

[CR95] Wyatt T, Rockich-Winston N, Taylor T, White D (2020). What does context have to do with anything? A study of professional identity formation in physician-trainees considered underrepresented in medicine. Academic Medicine.

[CR96] Wyatt T, Rockich-Winston N, White D, Taylor T (2020). Changing the narrative”: a study on professional identity formation among Black/African American physicians in the US. Advances in Health Sciences Education.

[CR97] Yap M, Konrad A (2009). Gender and racial differentials in promotions: Is there a sticky floor, a mid-level bottleneck, or a glass ceiling?. Relations Industrielles/Industrial Relations.

[CR98] Yuracko K (2003). Perfectionism and Contemporary Feminist Values.

